# Empirical Categorization of Factors Affecting Online Consumer Behavior of Gen Z Regarding Newly Launched Technological Products and Moderating Impact of Perceived Risk

**DOI:** 10.3390/bs15030371

**Published:** 2025-03-15

**Authors:** Dimitrios Theocharis, Georgios Tsekouropoulos, Chryssoula Chatzigeorgiou, Georgios Kokkinis

**Affiliations:** Department of Organization Management, Marketing & Tourism, International Hellenic University, 57400 Thessaloniki, Greece; geotsek@ihu.gr (G.T.); cchatzigeorgiou@ihu.gr (C.C.); kokkinis@mkt.teithe.gr (G.K.)

**Keywords:** online consumer behavior, Generation Z, newly launched products, marketing of technological products, perceived risk

## Abstract

In previous years, studying consumer behavior was seen as important, but in today’s fast-changing market, with rapid technological advancements, understanding consumer behavior can be a key factor in a product’s success or failure. The aim of the current research was to investigate the factors that can influence the online consumer behavior of Generation Z, regarding technological products that have just been launched and are available to the public. To achieve this goal, a cross-sectional research study was conducted with a sample of 302 Generation Z consumers selected using convenience sampling and elements of systematic sampling. This research used a structured questionnaire with established measurement scales to explore different aspects of online consumer behavior. The questionnaire was based on variables identified from various consumer behavior theories and models. The results led to the identification of six groups of influencing factors on online consumer behavior, highlighting the importance of these factors in shaping online consumer behavior and showing the influence of perceived risk as a moderating factor. These findings provide a thorough understanding of the factors that influence online consumer behavior while simultaneously laying the foundation for the creation of targeted and differentiated marketing strategies.

## 1. Introduction

Online consumer behavior can be shaped or influenced by a multitude of factors (Lichev, 2017; Patel & Chauhan, 2024; Nyrhinen et al., 2024; Chu, 2024), which may either stem from a company’s efforts to sell its products (e.g., promotional activities), arise as a consequence of the overall business strategy over time (e.g., building brand identity and customer engagement), or be linked to the consumer’s own personality traits (Theocharis & Tsekouropoulos, 2022). Predicting this behavior has the potential to lead to specific business decisions aligned with the company’s goals, usually with the ultimate aim of maximizing revenue and minimizing the likelihood of product failure (Kolanska-Stronka & Singh, 2024). In general, the introduction of a new product into the market requires significant expenditure in financial and human resources, along with ongoing support from the company (Oke et al., 2024). As a result, potential failure has substantial and serious consequences, not only for current profitability but also for the company’s overall sustainability. Therefore, reducing the possible risk associated with such endeavors is essential, ensuring the product’s market position and the achievement of business objectives. In this context, particularly when the products in question are technological in nature, different consumer groups tend to exhibit variations in their behavior (Bandara & Liyanage, 2024). Familiarity with technology often acts as a differentiating factor, which can sometimes complicate the extraction of reliable conclusions, since different levels of familiarity may lead to different product choices (Tamilmani et al., 2021). Thus, focusing on a consumer group with a high level of interaction with technology and the internet can lead to clearer insights and more effective marketing strategies. Generation Z (individuals born between 1995 and 2012) is characterized by its high dependency on technology, strong connection to the digital environment through various means (social media, mobile devices), frequent sharing of personal information online, aversion to complex messages, preference for direct advertising messages, and the tendency to conduct most of its purchases online (Purwanto, 2023; Jusuf, 2023; Ghosh et al., 2024). The goal of this research is to examine the factors influencing the online consumer behavior of Generation Z regarding newly released technological products. Additionally, it seeks to categorize the factors affecting consumer behavior and to explore the moderating role of perceived risk in the relationship between these factors and consumer behavior. Previous research efforts have often focused on identifying factors that influence consumer behavior but typically in isolation or to a limited extent in combination. Moreover, based on their conceptual frameworks and their actual meaning or definitions, these factors are often divided into different categories such as economic, behavioral, cultural, and social factors (Ramya & Ali, 2016; Dangi et al., 2020; Rodrigues et al., 2021; Etuk et al., 2022). This categorization, largely reliant on the subjective interpretation of each researcher, is not always grounded in empirical data derived from consumer behavior studies. Consequently, beyond examining the consumer behavior of Generation Z and the factors influencing it, this study will aim to categorize these factors based on the results of statistical analysis, offering a new typology. Finally, while the literature on new-to-market products often focuses on the pre-launch stage and their design (Francis & Hoefel, 2018; Grigoreva et al., 2021; Salam et al., 2024), this study will add knowledge about the factors influencing consumer behavior after the products have been introduced to consumers. This perspective provides a novel dimension that can help reduce the likelihood of failure for such new market entries.

### 1.1. Gen Z and Consumer Behavior

Generation Z constitutes the largest proportion of the global population and is simultaneously the most diverse generation to date (Walters, 2021; Van den Bergh et al., 2024). In this context, the key characteristics of Generation Z include growing up reading and writing reviews and having extensive knowledge of various information sources and easily switching between them. These factors are essential when trying to engage with Gen Z, and they strongly shape their consumer behavior, both before and after a purchase (Matendawafa & Farhangpour, 2016; Dabija et al., 2019; Afshan et al., 2022; Maziriri et al., 2023). As a generation deeply integrated with the digital world, Gen Z is influenced by a wide range of opinions, including those of friends, peers, and influencers, as well as reviews and ratings found on various online platforms (Tariyal et al., 2020).

Online reviews serve as sources of credibility and authenticity, significantly impacting Gen Z consumers’ purchase intentions, their trust in a brand, and their loyalty intentions toward that brand (Duffett, 2020; Thangavel et al., 2022; Sudirjo et al., 2023; Elkhwesky et al., 2024). Additionally, their social environment amplifies the desire for social acceptance, driving purchasing decisions that reflect the preferences and values of the group to which they belong (Vajkai & Zsoka, 2020; Serravalle et al., 2022; Zaib Abbasi et al., 2023). The study of Generation Z’s consumer behavior is of vital importance for businesses and researchers due to this generation’s increasing influence on global markets and its unique characteristics (Vajkai & Zsoka, 2020). Generation Z has grown up in an era entirely different from previous generations, particularly in terms of the role digital devices play in their lives and the importance of social issues, such as environmental harm (Cagala & Babcanova, 2024). As information technology has rapidly evolved, significant changes have also occurred regarding generational consumer behavior (Dragolea et al., 2023). Generation Z exhibits numerous differences compared to previous generations, particularly in communication, interaction, values, and priority setting (Priporas et al., 2017; Bratina & Faganel, 2024). Additionally, as they are still maturing, their ideas are constantly evolving. Therefore, understanding the characteristics and factors that shape and influence Generation Z’s consumer behavior is a highly significant area of research. This knowledge can determine the selection of marketing strategies and the development of product and service offerings in the future (Pitanatri et al., 2024). Businesses must be able to effectively approach this generation to maintain their connection with the consumer base, given that Generation Z is a rapidly evolving demographic (Ghosh et al., 2024). To sustain their viability in the future, businesses need to have the knowledge and readiness to adapt their offerings, aiming either to gain or maintain a competitive advantage in the market (Elkhwesky et al., 2024).

Generation Z primarily interacts with brands through online platforms, reshaping the way companies approach marketing, consumer engagement, and service delivery (Popa et al., 2023). The emphasis they place on values such as sustainability, inclusion, and authenticity drives businesses to adapt and innovate to meet Generation Z’s expectations (Muhammad et al., 2024). Furthermore, Generation Z represents 25–30% of consumers worldwide in 2024, making it the largest demographic group in terms of size (Shahid & Ikram, 2024). Additionally, Generation Z’s actual purchasing power is estimated at 360 billion dollars (Subawa et al., 2020). As Generation Z enters the workforce, moving from entry-level to higher-level positions with higher salaries, its income will increase, expanding its purchasing power (Hinduan et al., 2020). Ignoring these facts at the business level could significantly impact the sustainability and future growth of all kinds of businesses (Muhammad et al., 2024). Therefore, for businesses to achieve sustainability, focusing on Generation Z at both the research and business levels must be a fundamental objective, providing valuable information and data to all stakeholders involved (Siddiqui et al., 2022). In addition, combined with its purchasing power, Generation Z’s importance is enhanced by its influence on family and household decisions, making it a significant driver of consumption trends (Sharma & Kanchwala, 2022). Understanding the preferences and behavior of this generation helps businesses develop targeted strategies that address its needs while simultaneously predicting future market changes (Ling et al., 2024). Information on Generation Z’s preferences also provides a guide to leveraging emerging digital trends, such as social commerce and influencer marketing, ensuring relevance in a rapidly evolving consumer environment (Popa et al., 2023).

### 1.2. Newly Launched Products

The introduction of new products is critical to the success and survival of many businesses (Salmen, 2021; Fabo et al., 2023). However, predicting whether new products will be successful is one of the toughest challenges faced by managers, with failed attempts often resulting in enormous costs (Goodwin et al., 2014; Saha, 2019). Nevertheless, even if the forecast for a new product prior to its market introduction is positive, various factors can change the outcome. The consumer behavior of Generation Z in relation to new products, particularly technological products, represents an interesting area of study, as the preferences and habits of this generation significantly shape market trends and the success of innovations (Grigoreva et al., 2021). Regarding new incoming products, Generation Z shows a strong preference for innovation and freshness, actively seeking products that reflect the latest developments and align with their fast-paced and interconnected lifestyle (Thangavel et al., 2022). This is particularly true for technological products such as mobile devices, wearables, smart home devices, and gaming technology, which meet their needs for functionality, convenience, and integration into their digital activities (Erwin et al., 2023).

The purchasing decisions of Generation Z are strongly influenced by various key factors, such as their dependence on social media, peer recommendations, and endorsements from influencers (Salam et al., 2024). For new incoming products, these digital channels serve as a crucial means for enhancing brand recognition and promoting initial engagement (Sjahruddin & Adif, 2024). Social media platforms function as hubs for product discovery, where Generation Z consumers are exposed to creative marketing campaigns, videos, and detailed reviews (Roberts et al., 2017). These experiences often guide their first impressions and inform their perceptions of a product’s value and significance (Francis & Hoefel, 2018). Moreover, Generation Z consumers are known for their desire for personalized and authentic interactions with brands (Beregovskaya & Grishaeva, 2020). When evaluating new incoming products, they place great emphasis on experiences that appear tailored to their needs and values (Kushwaha, 2021). As mentioned, sustainability and ethical practices play a significant role in their purchasing decisions, as Generation Z consumers prefer brands that demonstrate commitment to environmental protection and social responsibility, with brands launching new products often finding success when showcasing these values in their messaging (Sjahruddin & Adif, 2024).

Generation Z’s preference for convenience and immediate gratification also influences the way they interact with new incoming products (Francis & Hoefel, 2018). These consumers expect seamless online shopping experiences, fast delivery, and easy access to customer support (Erwin et al., 2023). This generation is less tolerant of brands that fail to meet these expectations, as their loyalty often depends on continuous and high-quality interaction (Beregovskaya & Grishaeva, 2020). Additionally, the use of online word-of-mouth communication amplifies the impact of a consumer’s first experience (Thangavel et al., 2022). Positive feedback shared through online reviews, forums, or social media posts can significantly accelerate the adoption of a new incoming product, while negative experiences can just as easily diminish it (Dragolea et al., 2023). Finally, Generation Z’s trend toward innovation and exclusivity enhances their interest in participating in product launches (Grigoreva et al., 2021). In the field of technology products, limited releases and early access offers create a sense of exclusivity, which also appeals to Generation Z’s “fear” of missing out on something (Kushwaha, 2021).

### 1.3. Factors Affecting Online Consumer Behavior

**Figure 1:**
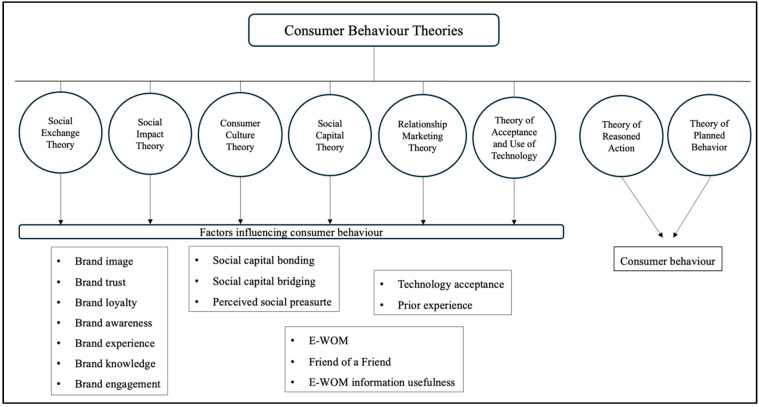
Initial theoretical model.

The study of consumer behavior has produced several theoretical approaches and models, often referred to as behavioral models or theories, aimed at understanding consumer behavior. This research, aiming among other things at a holistic study of consumer behavior and analyzing various theories, identified a number of factors that can determine consumer behavior online. The development of the theoretical framework that supported the research model combines multidimensional theories that offer a comprehensive understanding of the factors influencing consumer behavior, directly linking them to measurable variables. These theories include Social Exchange Theory, Social Impact Theory, Consumer Culture Theory, Social Capital Theory, Theory of Reasoned Action, Relationship Marketing Theory, Theory of Planned Behavior, and the Unified Theory of Acceptance and Use of Technology. Social Exchange Theory posits that human relationships, including consumer interactions, are based on a cost–benefit analysis where individuals seek to maximize rewards while minimizing costs (Bagozzi, 1975; Zhao & Detlor, 2023). In consumer behavior, this theory explains why customers remain loyal to brands that offer greater value compared to competitors (K. Wang et al., 2023; Oklevik et al., 2024). Similarly, Social Impact Theory suggests that individuals’ attitudes and behaviors are influenced by the strength, immediacy, and number of people in their social environment (Latane, 1996; Handarkho, 2021). This theory plays a crucial role in marketing, where peer influence, reviews, and social media endorsements significantly affect purchasing decisions (Afshan et al., 2022; Chavadi et al., 2023; Zeqiri et al., 2024). Consumer Culture Theory (CCT) explores how cultural influences shape consumption patterns and brand preferences (Hungara & Nobre, 2021). It highlights how consumers use products and brands to express identity, social status, and lifestyle, reinforcing the idea that marketing strategies must align with cultural values and trends (Kozinets & Jenkins, 2022; Thompson et al., 2023; Moorlock et al., 2023). Social Capital Theory, on the other hand, emphasizes the role of social networks and trust in facilitating consumer transactions (Kasim et al., 2022). Consumers are more likely to engage with brands recommended by trusted networks, such as family, friends, or online communities (Dhar & Bose, 2023; Dhar et al., 2024). The Theory of Reasoned Action (TRA) asserts that a consumer’s intention to purchase a product is determined by their attitudes and subjective norms (Ajzen, 2020). This means that if a person believes that buying a product aligns with their values and is socially approved, they are more likely to proceed with the purchase. Expanding on this, Relationship Marketing Theory focuses on building long-term relationships with customers rather than just driving immediate sales (Wongkitrungrueng et al., 2020). Loyalty programs, personalized experiences, and excellent customer service are strategies that brands use to enhance customer retention and advocacy. The Theory of Planned Behavior (TPB) extends TRA by incorporating perceived behavioral control, suggesting that consumers are more likely to act on their intentions if they feel they have the ability to do so (Ajzen, 1991, 2020). This is particularly relevant in online shopping, where ease of navigation and secure payment options can influence purchase behavior (Pillai et al., 2022; Khan et al., 2023). Lastly, the Unified Theory of Acceptance and Use of Technology (UTAUT) explains how various factors, such as performance expectancy, effort expectancy, social influence, and facilitating conditions, affect a consumer’s willingness to adopt new technologies (Venkatesh et al., 2016). This is especially relevant in e-commerce and digital marketing, where brands must ensure that new technologies are user-friendly and perceived as beneficial by consumers (Pramudito et al., 2023). Together, these theories provide a comprehensive understanding of how psychological, social, and technological factors drive consumer behavior, allowing marketers to develop more effective engagement strategies. Each theory is linked to specific variables that significantly influence consumer behavior (Figure 1).

**Figure 2:**
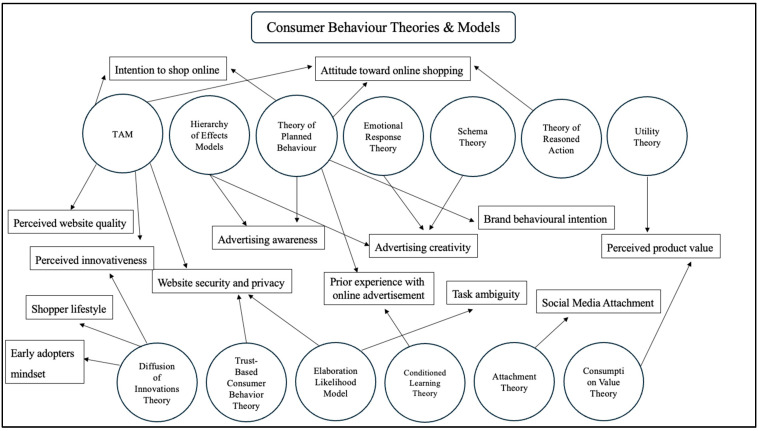
Highly influential factors of consumer behavior.

This multidimensional framework provides a comprehensive approach to studying consumer behavior by integrating various theoretical and practical variables. Additionally, as previously mentioned, the initial theoretical model (Figure 1) will be expanded to include additional variables, such as behavioral and attitudinal factors, marketing and advertising influences, online consumer experiences, and characteristics specific to Generation Z. These elements, referred to as highly influential factors, significantly shape consumer behavior by interacting both with each other and with the core components of the theoretical model. These factors can originate from the individual (such as personal traits or past actions) or from external brand influences. Rather than functioning independently, they interact dynamically, creating a complex and ever-evolving consumer behavior model that varies across individuals and contexts. Analyzing these factors both separately and in combination, particularly in relation to online consumer behavior and emerging technological products, will contribute to a more holistic model. This, in turn, will offer valuable insights to researchers and marketing professionals, aiding in more informed decision-making. This model will enable the prediction and shaping of strategies to influence online consumer behavior, taking into account the broader influence system that consumers are subjected to, rather than focusing on a behavior as the result of a single action or the influence of one or two factors. Specifically, highly influential factors show connections with one or multiple consumer behavior theories that explain and support their importance in creating a holistic model for predicting online consumer behavior regarding newly introduced technological products (Figure 2). The Technology Acceptance Model (TAM) explains that consumers adopt new technologies based on perceived usefulness and ease of use (Na et al., 2022; Solomovich & Abraham, 2024), which directly impacts their intention to shop online (J. B. Kim, 2012; Wibasuri et al., 2024). This concept ties into the Diffusion of Innovations Theory, which explains how new technologies and shopping behaviors spread (Basileo & Lyons, 2024), particularly among early adopters (Seebauer, 2015) with a specific shopper lifestyle (Ali et al., 2019). Similarly, the Trust-Based Consumer Behavior Theory reinforces the TAM by emphasizing the importance of website security and privacy (D. J. Kim et al., 2008; Benson et al., 2015), as trust is a key factor in technology adoption (L. Wang et al., 2014). The Hierarchy of Effects Model, which outlines how advertising influences consumer decision-making through awareness, interest, desire, and action (Kite et al., 2018), is closely linked to the Elaboration Likelihood Model (ELM), which differentiates between central and peripheral routes of persuasion, affecting prior experience with online advertisement (Lai & Huang, 2011). Both of these models play a role in advertising awareness and creativity, which influence consumers’ attitude toward online shopping as explained by the Emotional Response Theory (Horan et al., 2012). Additionally, Schema Theory suggests that consumers process new shopping experiences based on prior knowledge (Halkias, 2015), reinforcing the Theory of Planned Behavior (TPB) and Theory of Reasoned Action (TRA), which argue that consumer intentions are shaped by attitudes, subjective norms, and perceived behavioral control (Ajzen & Schmidt, 2020). These theories collectively impact brand behavioral intention (Dutta & Singh, 2014), influencing whether consumers engage with a particular brand. Meanwhile, the Utility Theory suggests that consumers make decisions to maximize perceived product value (Tanrikulu, 2021), which is strongly affected by social media attachment—a concept supported by Attachment Theory (Shorter et al., 2022) and Consumption Value Theory, which explain how emotional connections with brands and perceived benefits shape consumer loyalty and engagement (Kaur et al., 2018). Lastly, Conditioned Learning Theory connects to advertising and persuasion models by explaining how repeated exposure to marketing messages can either clarify or increase task ambiguity in consumer decision-making (Kitchen et al., 2014; Petty et al., 2017). These theories combined create a dynamic and interconnected framework that explains how cognitive, emotional, social, and technological factors influence consumer behavior, particularly in online shopping environments. In conclusion, nineteen (19) consumer behavior theories and models were assessed and analyzed to provide the theoretical foundation of this study and the variables—key influential factors—that will be measured for their impact on consumer behavior.

### 1.4. Importance of Perceived Risk

The choice of perceived risk as a moderating factor in the relationship between the variables under examination and consumer behavior is based on its strong influence on decision-making processes and consumer perceptions (Singh & Srivastava, 2018). Perceived risk significantly impacts consumer behavior, shaping willingness to purchase and the adoption of new products or services (Baidoun & Salem, 2024). High perceived risk can lead consumers to rely on critical trust-building elements, such as brand image, warranties, or third-party reviews, before making decisions (Sun et al., 2023). For instance, in e-commerce, consumers with high perceived risk may avoid purchasing from unfamiliar brands or using unknown platforms, preferring well-established names that offer security and reliability (Aufa & Marsasi, 2023). Conversely, consumers with low perceived risk are more likely to experiment with new products or brands, relying less on factors such as brand image or reviews (Sawang et al., 2023). By incorporating perceived risk as a moderating factor, marketers can better understand the diverse ways in which consumers respond to key influencing factors, enabling the development of targeted strategies that address the unique needs and expectations of different consumer groups.

**Figure 3:**
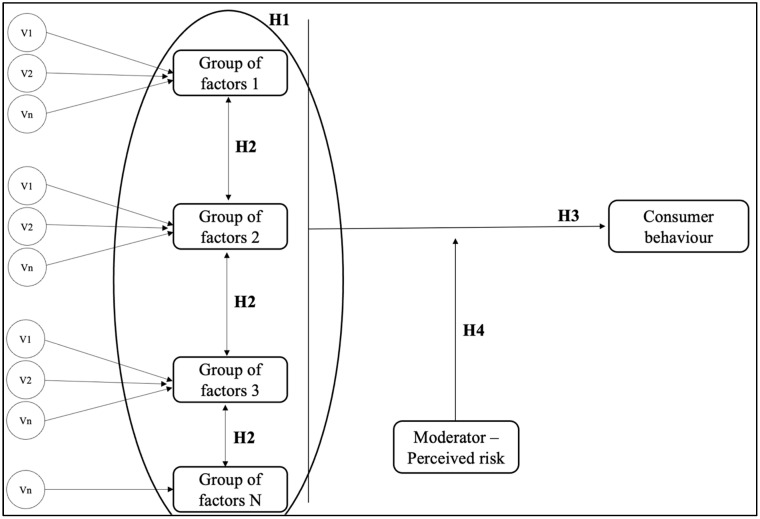
Research model.

In conclusion, this research aims to provide new findings and insights into the understanding of online consumer behavior, newly launched technological products in the market, Generation Z, and the existence of moderating variables. Based on this, the following research hypotheses were formulated, which will be answered through the research process and are depicted in the research model below (Figure 3).

**H_1_:**  *The variables that influence the online consumer behavior of Generation Z concerning newly launched technological products can be grouped into influencing factors.*

**H_2_:**  *The groups of influencing factors of the online consumer behavior of Generation Z concerning newly launched technological products show statistically significant correlations among themselves and with online consumer behavior.*

**H_3_:**  *The groups of influencing factors can predict the online consumer behavior of Generation Z concerning newly launched technological products.*

**H_4_:**  *Perceived risk acts as a moderating factor in the relationship between the groups of influencing factors and the online consumer behavior of Generation Z concerning newly launched technological products.*

## 2. Materials and Methods

### 2.1. Research Method

Consumers’ behavior as a research subject was approached through qualitative, quantitative, and mixed research methods. In the present study, a quantitative research methodology was employed to produce measurable and comparable results. This approach is characterized as cross-sectional research which is typically used to explore relationships between variables or to describe the characteristics of a population, such as demographics, behavior, or attitudes (Maier et al., 2023).

### 2.2. Sampling and Participants

In the present study, the target population was Generation Z, focusing on individuals born between 1997 and 2012, who represent a digitally savvy, technologically experienced, and influential consumer group. However, due to the difficulty of obtaining consent from legal guardians, all individuals under the age of 18 within Generation Z were excluded, which constitutes a research limitation for studying this generation. The research sample included 302 participants, exceeding the minimum sample size determined through G-Power v.3.1.9.6 analysis (to achieve a power of 0.80 in a test with a significance level of α = 0.05 and medium-to-large effect sizes). The analysis estimated a range of 160–180 participants as sufficient for the planned statistical analyses. The final sample consisted of undergraduate students from various disciplines who study at a Greek university and born within the range 1997 to 2012. The sampling method primarily used was convenience sampling, supplemented by elements of systematic sampling at certain stages of the process to enhance representativeness (Marczyk et al., 2010; Saunders et al., 2019). Specifically, the entire sample was selected due to the researcher’s access to it. Nevertheless, participants were chosen from a sampling frame where available population units were recorded without a specific order to avoid potential bias from alphabetical sorting, with selection occurring at odd-numbered positions (1, 3, 5, 7, etc.). Convenience sampling was chosen for its practical advantages, such as accessibility, efficiency, and the ability to quickly gather responses from a population with a strong presence in digital environments like social networks and online platforms. This method aligns with Generation Z’s high online activity (Munsch, 2021), making it both cost-effective and suitable for the present research. Finally, the inclusion of systematic sampling elements, as described above, in certain stages of the process adds rigor, reducing potential bias and enhancing the reliability of the findings. This hybrid approach ensures that the sample adequately reflects the diversity of behaviors and attitudes within Generation Z, making it a robust choice for exploring consumer behavior patterns in a digitally oriented and rapidly evolving demographic group. Table 1 shows the answers of the respondents regarding their demographic characteristics. Specifically, 52.3% of the respondents were women, 85.4% were university students, 41.7% had a family income between EUR 10,000 and 20,000, and the average age of the sample was 20.52 years (SD = 2.35).

### 2.3. Research Tool and Data Collection

The research tool for this quantitative study was a structured questionnaire designed to explore various dimensions of consumer behavior, particularly in relation to the purchase of new technological products by Generation Z. The questionnaire consisted of seven sections, each focusing on specific aspects of participants’ demographic characteristics, purchasing behavior, and perceptions, with the aim of providing a concise presentation to respondents. A pilot version of the questionnaire was used to refine the wording of the questions to ensure clarity and relevance, thereby guaranteeing high-quality data collection. During the pilot phase, discussions with participants were conducted to assess whether the questions were understandable, relevant, and appropriate. The questionnaire included demographic variables such as age, gender, education level, and family income. It explored the purchase of new technological products, with questions addressing the type of product, brand, online purchasing behavior, location of the online store, product type (low or high involvement, luxury), degree of innovation, ease of purchase (task ambiguity), respondents’ early adopters mindset, website characteristics (quality, security, and privacy), the influence of electronic advertising (recognizability and creativity), perceived product value, and various brand attributes, including brand experience, loyalty, trust, knowledge, behavioral intention, awareness, and image. Social media usage was also investigated, focusing on years of use, frequency, and daily time spent. This study examined shopping lifestyles through questions about respondents’ purchasing habits and delved into online purchasing behavior, including previous online shopping experiences, attitudes towards online shopping, acceptance of the internet as a technology, perceived risk, and the intention to make online purchases. The influence of others was another focus, with questions about perceived social pressure, online word-of-mouth communication, the usefulness of such communication, interaction from friends of friends, and social capital. Consumer behavior was addressed through questions about purchase intention, actual purchases, post-purchase behavior, post-purchase loyalty intention, and the unique characteristics of Generation Z. The questionnaire used 5- or 7-point Likert scales, with verbal anchors, such as “Strongly Disagree–Strongly Agree” or “Not at all–Very much”, to measure perceptions, attitudes, and behavioral intentions, as well as dichotomous scales where necessary. The data collected using this comprehensive tool were employed for detailed statistical analyses, providing insights into consumer behavior patterns in the context of purchases of new-to-market technology products. A key advantage of the tool was the use of well-established scales, widely utilized in numerous studies to ensure validity and reliability (Table 2).

The questionnaire incorporated these established scales to ensure the reliability of responses in key dimensions such as technology adoption, consumer trust, and purchasing behavior. A rigorous pilot test refined the wording to enhance clarity and cultural relevance. Following data collection, strict validation procedures were applied to assess the validity and reliability of the scales and the tool (including confirmatory factor analysis, outlier detection, reliability testing, and normality checks) (Field, 2024). Confirmatory Factor Analysis (CFA) was employed to verify the structure of the scales, ensuring they measured the intended constructs. Reliability analysis evaluated the consistency of the scales, while outlier detection and normal distribution checks ensured the statistical integrity of the data. Once the data were deemed reliable and valid, both descriptive and inferential statistical analyses were conducted to test the research hypotheses, providing reliable insights into the phenomena under study. This comprehensive approach ensures the validity of the tool, addressing both academic rigor and practical applicability. Descriptive statistical analysis, presented through charts and tables, provided an overall view of the data, capturing the sample’s responses across all questions. Inferential statistical analysis, including correlation analysis, multiple regression, and moderation analysis, was used to test the research hypotheses. Finally, this study adhered to established ethical guidelines, as outlined in the Declaration of Helsinki, ensuring voluntary participation and informed consent while protecting participants’ privacy and confidentiality (Ashcroft, 2008).

## 3. Results

### 3.1. Grouping the Factors Influencing Online Consumer Behavior

Factor analysis was used to group the factors influencing consumer behavior. Initially, the Kaiser–Meyer–Olkin (KMO) measure was 0.711, indicating moderate sampling adequacy. Bartlett’s Test of Sphericity was statistically significant (χ^2^ = 2639.450, df = 253, *p* < 0.001), indicating that the correlations among the variables were sufficiently high to justify factor analysis. Overall, five factors were extracted, explaining 66.562% of the total variance (Table 3).

The loadings for the variables range from 0.412 to 0.882, indicating that most variables are well represented in the extracted factors. The variables that comprise each factor are shown in Table 4 below.

The main factors include behavioral and attitudinal factors, social and peer influences, marketing and advertising impact, online experience, brand-related factors, and Gen Z characteristics (Table 5). The last factor was added separately to the analysis, formed based on characteristics of Generation Z derived from the literature. Overall, the results provide a solid understanding of the factors influencing consumers’ perceptions and behaviors in relation to online shopping behavior.

### 3.2. Correlations of Influencing Factors and Online Consumer Behavior

Table 6 presents the correlations between the groups of factors influencing consumer behavior. All correlations are significant at the 0.01 level, indicating strong or moderate relationships between some variables. The strongest correlation is observed between “behavioral and attitudinal factors” and “brand-related factors” (r = 0.656, *p* < 0.01), suggesting that behavioral and emotional factors significantly influence Gen Z’s attitudes and perceptions of brands. Additionally, “behavioral and attitudinal factors” show a high correlation with “social and peer influences” (r = 0.646, *p* < 0.01), indicating that social influences are critical in shaping consumer behavior. Notably, there is also a moderate correlation between “social and peer influences” and “marketing and advertising impact” (r = 0.430, *p* < 0.01), highlighting the role of marketing in reinforcing social influences. The “online experience” factor shows weaker correlations with other variables, indicating that while it plays a role, it is not as influential as other factors. Lastly, the factor related to Gen Z characteristics shows positive correlations with all factors, with the strongest being with “brand-related factors” (r = 0.414, *p* < 0.01), underscoring the importance of understanding Gen Z characteristics for effective branding strategies. Overall, the results demonstrate that social, behavioral, and brand-related factors are strongly interconnected, while online experiences have a relatively lower impact. These insights are valuable for creating targeted marketing strategies aimed at Generation Z. Additionally, all factors present positive correlations with online consumer behavior, indicating that, in general, all variables are expected to exert influence, with behavioral and attitudinal factors and social and peer influences showing the strongest correlations. Finally, the findings indicate the multidimensional nature of online consumer behavior and the multiple interactions between factors that can influence consumer behavior.

### 3.3. Regression Analysis for the Prediction of Online Consumer Behavior

Table 7 provides a summary of the prediction models for online consumer behavior. This table includes various statistics to assess the performance of the model. Model 1 has a correlation coefficient (R) of 0.746, indicating a strong relationship between the predictors and the dependent variable (online consumer behavior). The R Square value is 0.557, which means that approximately 55.7% of the variance in consumer behavior can be explained by the independent variables in the model. The Adjusted R Square value of 0.538 accounts for the number of predictors in the model, adjusting the R Square value to provide a more accurate representation. The standard error of the estimate is 0.46977, showing the average distance between the observed values and the predicted values. Finally, the Durbin–Watson statistic is 1.960, which is close to the ideal value of 2, suggesting that there is no significant autocorrelation in the residuals, implying that the model fits the data well.

Table 8 presents the coefficients of the prediction models for overall consumer behavior. The unstandardized coefficients show the magnitude of change in the dependent variable (online consumer behavior) for each unit change in the independent variables. The standardized coefficients (Beta) provide an understanding of the relative importance of each predictor, with “behavioral and attitudinal factors” (Beta = 0.267) and “social and peer influences” (Beta = 0.273) showing the strongest contributions. The t-values and *p*-values indicate the significance of each predictor, with “behavioral and attitudinal factors” (*p* = 0.010), “social and peer influences” (*p* = 0.003), “marketing and advertising impact” (*p* = 0.004), and “Gen Z characteristics” (*p* = 0.004) being statistically significant at the 0.05 level. The confidence intervals for B values provide the range within which the true value of each coefficient is likely to fall. Regarding collinearity statistics, the Tolerance values for all variables are above 0.1, and the VIF values are all below 5, suggesting that there is no significant multicollinearity among the predictors. Overall, the results show that “behavioral and attitudinal factors”, “social and peer influences”, “marketing and advertising impact”, and “Gen Z characteristics” are significant predictors of online consumer behavior.

Based on the final model, in which all factors are included as independent variables, the following regression equation was developed for overall consumer behavior. Specifically, for each unit increase in an independent variable, the dependent variable (online consumer behavior) increases by the amount of b, assuming all other variables remain constant.

Online Consumer Behavior = −1.210 + 0.348 × (Behavioral and Attitudinal Factors) + 0.380 × (Social and Peer Influences) + 0.191 × (Marketing and Advertising Impact) + 0.131 × (Online Experience) − 0.008 × (Brand-Related Factors) + 0.205 × (Gen Z Characteristics)

The results highlight the importance of “behavioral and attitudinal factors”, “social and peer influences”, and “marketing and advertising impact”, as well as the characteristics of Generation Z, in consumer behavior. Despite the contribution of other factors, the limited significance of certain variables in the model suggests the need for further research or a revision of the framework. Finally, it can be said that the model provides a strong foundation for understanding the complexity of Gen Z’s consumer behavior. Additionally, the prediction model can serve as a valuable marketing tool for practitioners, providing a basis for understanding the interconnection of these factors with online consumer behavior.

### 3.4. Moderation Effects of Perceived Risk

Perceived risk was used as a moderating factor in the relationship between the influencing variables and consumer behavior. Below are the cases where perceived risk affects the relationship between the variables.

#### 3.4.1. Brand-Related Factors and Online Consumer Behavior

In this moderation analysis, the effects of brand-related factors on online consumer behavior were examined, with perceived risk acting as a moderating variable. The overall model was statistically significant, with an R^2^ value of 0.2572, indicating that 25.72% of the variance in online consumer behavior is explained by the influencing factor “brand-related factors”, perceived risk, and their interaction (F_(3, 298)_ = 34.3876, *p* < 0.001) (Table 9).

In general, it appears that there is a statistically significant baseline level of overall consumer behavior when the brand-related factors (BRFs) and perceived risk are zero. Furthermore, it seems that perceived risk has a marginally significant negative direct effect on overall consumer behavior (Table 10).

#### 3.4.2. Interaction and Conditional Effects of “Brand-Related Factors and Online Consumer Behavior”—Perceived Risk

The significant interaction term (b = 0.1956, *p* = 0.0203) indicates that the relationship between brand-related factors and online consumer behavior is dependent on—moderated by—perceived risk (Table 11).

Moreover, at low levels of perceived risk, the relationship between brand-related factors and online consumer behavior is weaker but significant (b = 0.3212, *p* < 0.001). At moderate and high levels, the effect is significantly strengthened, with B increasing to 0.4367 and 0.5522, respectively, and remaining highly significant (*p* < 0.001) (Table 12).

**Figure 4:**
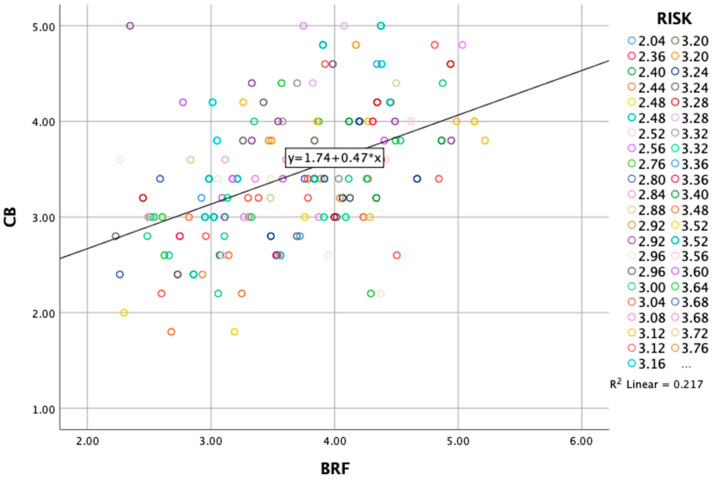
Brand-related factors and online consumer behavior—perceived risk.

The Johnson–Neyman technique identified the value of perceived risk (2.3141) below which the effects of brand-related factors on online consumer behavior are not significant and above which they become significant. In conclusion, the analysis reveals that while brand-related factors (BRFs) do not have a significant direct effect on online consumer behavior (CB), the relationship between them is significantly modified by perceived risk. Specifically, the effects of brand-related factors on online consumer behavior are significant when perceived risk is above 2.3141. As perceived risk increases, the positive effects of brand-related factors on online consumer behavior become stronger. For example, at a low risk level (2.8566), the effect is 0.3212, while at a high-risk level (4.0377), the effect increases to 0.5522. This significant interaction highlights that perceived risk plays a critical role in strengthening the relationship between brand-related factors and overall consumer behavior, suggesting that the more risk consumers perceive, the stronger the influence of brand-related factors on their online behavior (Figure 4). As a result, the findings highlight the importance of branding and consumers’ tendency to rely on it when they perceive that a buying decision involves high risk.

#### 3.4.3. Gen Z Characteristics and Online Consumer Behavior

In this moderation analysis, the effects of Generation Z characteristics (GZCs) on online consumer behavior were examined, with perceived risk acting as a moderating variable. The overall model was statistically significant, with an R^2^ value of 0.2711, indicating that 27.11% of the variance in online consumer behavior is explained by the influencing factor “Gen Z characteristics”, perceived risk, and their interaction (F_(3, 298)_ = 36.9406, *p* < 0.001) (Table 13).

In general, it appears that there is a statistically significant baseline level of online consumer behavior when Gen Z characteristics (GZCs) and perceived risk are zero. Furthermore, it seems that perceived risk has a marginally significant negative direct effect on online consumer behavior (Table 14).

#### 3.4.4. Interaction and Conditional Effects of “Gen Z Characteristics and Online Consumer Behavior”—Perceived RISK

The significant interaction term (b = 0.2110, *p* = 0.0115) indicates that the relationship between Gen Z characteristics and online consumer behavior is dependent on—modified by—the perceived risk (Table 15).

Subsequently, at low levels of perceived risk, the relationship between Gen Z characteristics and online consumer behavior is weaker but still significant (b = 0.3578, *p* < 0.001), while at moderate and high levels, the effect strengthens significantly, with b increasing to 0.4824 and 0.6071, respectively, and remaining highly significant (*p* < 0.001) (Table 16).

**Figure 5:**
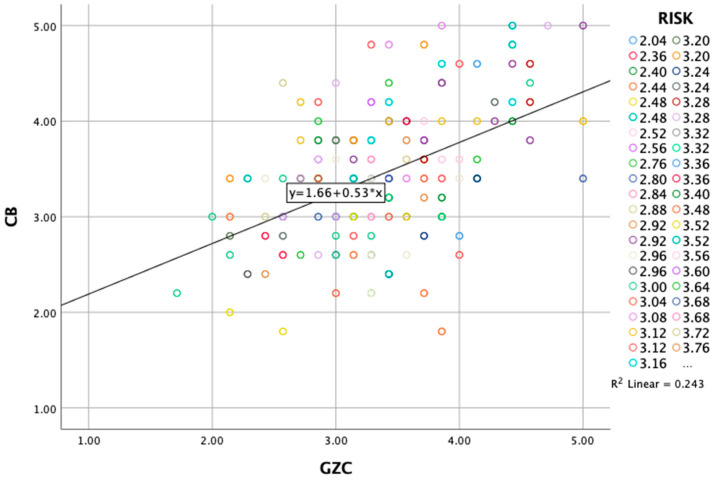
Gen Z characteristics and online consumer behavior—perceived risk.

The Johnson–Neyman technique identified the value of perceived risk (2.2626) below which the effects of Generation Z characteristics on overall consumer behavior are not significant and above which they become significant. In conclusion, the analysis reveals that while the influencing factor “Gen Z characteristics” does not have a significant direct effect on overall consumer behavior, the relationship between Gen Z characteristics and overall consumer behavior is significantly affected—modified—by perceived risk. Specifically, the effects of Gen Z characteristics on online consumer behavior are significant when perceived risk exceeds 2.2626. As perceived risk increases, this positive effect becomes stronger. For example, at a low risk level (2.8566), the effect is 0.3578, while at a high-risk level (4.0377), the effect increases to 0.6071. This significant interaction highlights that perceived risk plays a crucial role in strengthening the relationship between Gen Z characteristics and online consumer behavior, suggesting that as consumers perceive more risk, the influence of Gen Z characteristics on their behavior becomes more pronounced (Figure 5). As a result, the findings highlight the importance of Gen Z’s characteristics and consumers’ tendency to act based on them when they perceive a buying decision to involve high risk.

## 4. Discussion

The first research hypothesis was formulated to address the literature gap concerning the grouping of factors that influence consumer behavior in general and specifically for newly launched technological products in the market. The available literature on consumer behavior and the factors that shape it tends to categorize influencing factors mainly based on the content and definition of each factor, which often leads to subjective judgments and classifications with very broad boundaries. This research, through the first research hypothesis, which is supported by the research findings, concluded with the grouping of the factors that influence consumer behavior based on the data collected, distinguishing the influencing factors into six categories or groups. Overall, these findings provide a good understanding of the factors that influence consumer perceptions and behaviors in relation to online purchasing behavior and offer, based on the entirety of the analysis, an opportunity to form differentiated marketing and communication strategies for every marketing professional, particularly for companies dealing with new technological products. These specific differentiated strategies are capable of reducing the likelihood of failure and, to the extent possible, ensuring the success of a newly launched product in the market. Additionally, this very significant finding adds a distinct categorization of the factors influencing consumer behavior based on empirical data, which contrasts with classifications like those of Ramya and Ali (2016) and Dangi et al. (2020), who categorize the factors shaping consumer behavior based on their meaning and/or conceptual content. Overall, the first research hypothesis is supported by the findings of this study.

Regarding the second research hypothesis, the findings provide valuable insights into how different groups of factors interact to shape consumer behavior. The results from the analysis offer a comprehensive understanding of the factors that influence overall online consumer behavior in relation to newly launched technological products, highlighting the interaction between behavioral and social factors and factors related to the brand, marketing, and online experience. Behavioral and attitude-related factors, such as the intention behind online purchases and technology acceptance, emphasize the importance of creating a consumer-friendly technological environment and promoting positive attitudes towards online shopping. These findings, in agreement with the existing literature (J. B. Kim, 2012; Ha & Nguyen, 2010; Sutisna & Handra, 2022; Zhu et al., 2023; Higueras-Castillo et al., 2023; Wibasuri et al., 2024), form the basis for shaping consumer engagement and behavior, as they show that the willingness to adopt technology and participate in e-commerce significantly enhance online purchasing activities. Factors related to the social environment and peer influence highlight the critical role of interaction between individuals and communities in shaping consumer behavior, as noted in the existing literature (Tsekouropoulos, 2019; Al-Gasawneh & Al-Adamat, 2020; Bu et al., 2021; Siripipatthanakul et al., 2022; Jain et al., 2023). Furthermore, the influence of marketing and advertising underscores the need for creative and appealing communication strategies aimed at influencing consumer behavior, a point supported by numerous studies (Chavadi et al., 2020; Jinadasa et al., 2020; Choudhary, 2021; Amad et al., 2022; Shamim & Islam, 2022; Kurdi et al., 2022). Brand-related factors emerge as critical determinants of overall consumer behavior, with variables such as brand knowledge, brand loyalty, and brand image showing strong positive correlations with overall consumer behavior. These findings reinforce the importance of brand-building efforts to enhance trust and loyalty while simultaneously confirming the existing literature regarding branding and its influence on consumer behavior (Cropanzano & Mitchell, 2005; Sierra & McQuitty, 2005; Rather & Hollebeek, 2019; Farhana, 2021; Hoang, 2022; Parris & Guzman, 2023; Cai et al., 2023; Wong et al., 2023; Pham et al., 2023; K. Wang et al., 2023; Behl et al., 2024; Lee et al., 2024; Song et al., 2024).

Overall, the research findings provide valuable insights into the multidimensional factors influencing overall online consumer behavior. Behavioral and attitude-related factors serve as a foundation for consumer engagement, while social influences reinforce these behaviors through trust and community dynamics. Marketing and advertising-related factors play a critical role in shaping perceptions and capturing attention, particularly when they involve creativity. Additionally, the online experience itself, with elements such as security and familiarity, is a key factor in building trust and willingness to participate in e-commerce. Finally, brand-related factors highlight the ongoing importance of brand loyalty, image, and knowledge in shaping the behavior of Generation Z consumers.

Regarding the third research hypothesis, which is partially accepted, the results indicate that Generation Z’s consumer behavior can be effectively predicted, particularly by focusing on social, behavioral, and marketing-related influencing factors, as well as Generation Z’s characteristics. These factors interact to form a predictive model that does not include brand-related factors and online experience, which have been found to individually influence consumer behavior. These results align with numerous studies that discuss the individual influence of behavioral and attitude factors (J. B. Kim, 2012; Ha & Nguyen, 2010; Sutisna & Handra, 2022; Zhu et al., 2023; Higueras-Castillo et al., 2023; Wibasuri et al., 2024), social influences (Tsekouropoulos, 2019; Al-Gasawneh & Al-Adamat, 2020; Bu et al., 2021; Siripipatthanakul et al., 2022; Jain et al., 2023), marketing and advertising (Chavadi et al., 2020; Jinadasa et al., 2020; Choudhary, 2021; Amad et al., 2022; Shamim & Islam, 2022; Kurdi et al., 2022), and Generation Z’s characteristics (Dabija et al., 2019; Djafarova & Bowes, 2021; Djafarova & Foots, 2022; Krippes et al., 2024; Pitanatri et al., 2024) on consumer behavior. In contrast, the findings contradict other research efforts that highlight the impact of brand-related factors (Cropanzano & Mitchell, 2005; Sierra & McQuitty, 2005; Rather & Hollebeek, 2019; Farhana, 2021; Hoang, 2022; Parris & Guzman, 2023; Cai et al., 2023; Wong et al., 2023; Pham et al., 2023; K. Wang et al., 2023; Behl et al., 2024; Lee et al., 2024; Song et al., 2024) and online experience (Akasreku et al., 2023; Suganda & Arrifianti, 2023; Zhou & Liu, 2023) on consumer behavior.

Finally, the analysis of perceived risk as a moderating variable revealed significant variations in consumer behavior, which affect marketing strategies for newly launched technological products. Specifically, in high-risk situations, consumers are more likely to turn to well-known and reliable brands, as this reduces the uncertainty surrounding the purchasing decision. This finding is consistent with the research by Sun et al. (2023) and Aufa and Marsasi (2023), who state that high perceived risk can lead consumers to rely on important trust-building factors, such as brand image, warranties, third-party reviews, and their knowledge of a brand, to maximize safety and reliability. Therefore, despite aligning with these studies, the results of this research did not find any differences in the influence of the social environment-related factor on consumer behavior, according to the level of perceived risk. At the same time, given Generation Z’s characteristics, such as the search for innovation and connection with modern technology, this generation becomes more decisive in their purchasing decisions when perceived risk is high. This suggests that Generation Z consumers may be willing to take high risks if the product they are considering purchasing is innovative and technologically modern, which aligns with their tendency to try and adopt new technologies, as described in the literature (Djafarova & Bowes, 2021; Serravalle et al., 2022; Tata et al., 2023).

## 5. Conclusions

Through the analysis of the research data, this study developed a comprehensive and clear categorization of the factors that shape the online consumer behavior of Generation Z, distinguishing them into six main categories. These findings provide a thorough understanding of the factors that influence online consumer behavior while simultaneously laying a foundation for the creation of targeted and differentiated marketing strategies. These strategies can be tailored to the needs of each category of factors and applied by marketing professionals and businesses operating in the field of technological products. As a result, the risk of failure can be reduced, and the success of new products entering the market can be enhanced, providing a competitive advantage to businesses. Furthermore, the multi-level influence of all the groups of influencing factors and the variables within them on consumer behavior was highlighted, while the predictive model created indicated that the consumer behavior of Generation Z can be effectively predicted, particularly by focusing on social, behavioral, and marketing-related influencing factors, as well as the characteristics of Generation Z. Finally, the analysis of perceived risk as a moderating variable revealed that when Generation Z consumers perceive high risk regarding a purchase, the factors related to the brand and the characteristics of Generation Z seem to have a stronger influence on their consumer behavior.

### 5.1. Practical Implications

The distinction of influencing factors into six clear categories provides a comprehensive picture of the complexity that governs consumer behavior. The practical value of these findings is noteworthy and significant, as it forms the basis for developing differentiated marketing and communication strategies. These strategies, tailored to the specific needs and characteristics of Generation Z, can contribute to reducing the likelihood of failure and maximizing the success of new technological products in the market. Based on the research results, companies should focus on understanding and enhancing the behavioral and emotional factors that influence consumer behavior, leveraging brands as a means of communicating these values. Since the social environment plays a critical role, it becomes essential to utilize its influence through strategies that involve collaborations with communities, influencers, or participatory campaigns. At the same time, marketing and advertising should be integrated into a broader framework that combines social, emotional, and online experiences. Finally, adapting branding strategies to Generation Z’s preferences, especially through innovative approaches, can strengthen the connection with this generation. In conclusion, businesses’ marketing strategies should be adapted according to perceived risk, as consumers tend to rely more on the brand and communication strategies, which should focus on strengthening the consumer–brand connection, enhancing the characteristics of Generation Z, and reducing perceived risks through communication.

### 5.2. Research Limitations and Future Research

This research provides valuable insights into the factors that influence the consumer behavior of Generation Z regarding newly introduced technological products, but it is not without its limitations. Initially, the exclusion of participants under the age of 18, although ethically necessary, limits the scope of this study and omits the views of younger members of Generation Z, who are active consumers of technological products. Therefore, this research focused on part of the Generation Z consumer group, with the non-participating segment, about one-third, being a point of concern and potentially altering this study’s results. Furthermore, the use of convenience sampling, while practical, limits the generalizability of the findings, as the sample may not fully represent the diverse characteristics of the broader Generation Z population. Additionally, potential geographic limitations of the sample also reduce the applicability of the results in different cultural and socio-economic contexts.

Future research could extend this study by exploring several critical areas to deepen the understanding of Generation Z’s online consumer behavior. A key step would be to expand the participant base to include individuals under the age of 18, examining the entire Generation Z consumer group. Including teenagers could provide a more complete understanding of Generation Z, as their consumer behavior and attitudes may differ from those of the older members of the generational group. Furthermore, potential differences could be identified between younger and older consumers of Generation Z. The use of longitudinal studies is also recommended to track the evolution of attitudes, behavior, and purchasing patterns over time. These studies could offer insights into how external factors—such as economic conditions, technological advancements, and social changes—affect their consumer behavior. Long-term data monitoring could also reveal causal relationships and highlight generational shifts in values and purchasing motivations. Understanding how Generation Z adapts to new market conditions, such as an increased focus on sustainability or digital transformation, would be crucial for predicting their future behaviors. Additionally, cross-cultural studies comparing Generation Z’s behavior in different countries or regions could provide valuable information on how cultural, economic, and social factors shape consumer preferences.

## Figures and Tables

**Table 1:** Demographics.

Variable		Percentage
Gender	Male	47.7
	Female	52.3
Educational level	High school	0.7
	University student	85.4
	Bachelor’s degree	11.2
	Masters’ degree	2.7
Family income	<EUR 10,000	19.2
	EUR 10–20,000	41.7
	>EUR 20,000	39.1
Age	Mean	SD
	20.5298	2.35068

**Table 2:** Research scales.

Variables	
TAM (Davis, 1989; Lim & Ting, 2012; Juniwati, 2014)	Friend of a friend (Goyette et al., 2010; Filieri, 2015)
Shopper lifestyle scale (Huseynov & Ozkan Yıldırım, 2019)	Social media attachment (Own development)
Prior online experience (Johnson & Grayson, 2005)	E-WOM (Goyette et al., 2010; Filieri, 2015)
Task ambiguity (Jarvelainen, 2007)	Prior experience with online advertisement (Own development)
Perceived social pressure (Jarvelainen, 2007)	Advertising creativity (Yang & Smith, 2009)
Perceived brand innovativeness (Calantone et al., 2006)	Advertising awareness (Own development)
Perceived risk (Wai et al., 2019)	Brand awareness (Yoo et al., 2000)
Perceived product value (Sweeney & Soutar, 2001)	Brand trust (Johnson & Grayson, 2005; Kumar Ranganathan et al., 2013)
Website security and privacy (San Martin & Camarero, 2009)	Attitude towards online shopping (Lim & Ting, 2012)
Perceived website quality (McKnight et al., 2002)	Social capital bonding (Appel et al., 2016)
Brand behavioral intention (Rather & Hollebeek, 2019; Coudounaris & Sthapit, 2017)	Social capital bridging (Appel et al., 2016)
Online brand engagement (Rather & Hollebeek, 2019)	Brand knowledge (Hanaysha, 2016; Jin et al., 2012)
Online brand experience (Rather & Hollebeek, 2019; Tsaur et al., 2007)	Brand image (Hanaysha, 2016; Jin et al., 2012)
E-WOM information usefulness (Davis, 1989; Cheung & Thadani, 2012)	Brand loyalty (Hanaysha, 2016; Jin et al., 2012)
Intention to shop online (Lim & Ting, 2012)	Gen Z (Own development)
Early adopters mindset (Zijlstra et al., 2020)	Consumer behavior (Voramontri & Klieb, 2019; Chopra et al., 2020; Akayleh, 2021)

**Table 3:** KMO and Bartlett’s Test for grouping factors affecting online consumer behavior.

Kaiser–Meyer–Olkin Measure of Sampling Adequacy.		0.711
Bartlett’s Test of Sphericity	Approx. Chi-Square	2639.450
	df	253
	*p*.	0.000
Factors Extracted	5	
Variance Explained Factor 1	12.639%	
Variance Explained Factor 2	12.317%	
Variance Explained Factor 3	10.865%	
Variance Explained Factor 4	8.971%	
Variance Explained Factor 5	8.540%	
Total Variance Explained	66.562%	

**Table 4:** Rotated component matrix for grouping of factors influencing online consumer behavior.

	Comp. 1	Comp. 2	Comp. 3	Comp. 4	Comp. 5
Technology acceptance	0.820				
Attitude toward online shopping	0.782				
Brand behavioral intention	0.779				
Perceived brand innovativeness	0.713				
Intention to shop online	0.679				
Online brand engagement	0.586				
Shopper lifestyle	0.521				
Early adopters mindset	0.435				
Perceived social pressure		0.790			
E-WOM		0.790			
Social capital bridging		0.716			
E-WOM information usefulness		0.712			
Friend of a friend		0.602			
Social capital bonding		0.460			
Social media attachment		0.412			
Brand knowledge			0.882		
Brand loyalty			0.847		
Brand image			0.642		
Online brand experience			0.635		
Brand trust			0.625		
Brand awareness			0.539		
Perceived product value			0.472		
Website security and privacy				0.758	
Perceived website quality				0.745	
Task ambiguity				0.661	
Prior online experience				0.521	
Prior experience with online advertisement					0.834
Advertising awareness					0.764
Advertising creativity					0.587

Extraction method: principal component analysis.

**Table 5:** Distribution of variables among factors influencing online consumer behavior.

Behavioral and Attitudinal Factors	Social and Peer Influence Factors	Marketing and Advertising Impact Factors	Online Experience Factors	Brand-Related Factors	Gen Z Characteristics
Perceived brand innovativeness	Social media attachment	Prior experience with online advertisement	Task ambiguity	Perceived product value	Review dependency
Early adopters mindset	Perceived social pressure	Advertising creativity	Perceived website quality	Brand knowledge	Influencers’ impact
Online brand engagement	E-WOM	Advertising awareness	Website security and privacy	Brand image	Comment dependency
Brand behavioral intention	Friend of a friend		Prior online experience	Brand trust	Visual aspect dependency
Shopper lifestyle	E-WOM information usefulness			Brand loyalty	Sustainable image dependency
Attitude toward online shopping	Social capital bonding			Brand awareness	Price dependency
Technology acceptance	Social capital bridging			Online brand experience	Brand community dependency
Intention to shop online					

**Table 6:** Correlations between groups of factors influencing online consumer behavior.

		1	2	3	4	5	6	7
Spearman’s rho	1. Behavioral and attitudinal factors	1.000	0.646 **	0.356 **	0.312 **	0.656 **	0.396 **	0.626 **
	2. Social and peer influences		1.000	0.430 **	0.259 **	0.513 **	0.495 **	0.613 **
	3. Marketing and advertising impact			1.000	0.325 **	0.375 **	0.274 **	0.444 **
	4. Online experience				1.000	0.224 **	0.173 **	0.307 **
	5. Brand-related factors					1.000	0.414 **	0.486 **
	6. Gen Z characteristics						1.000	0.458 **
	7. Online consumer behavior							1.000

** Correlation is significant at the 0.01 level (2-tailed).

**Table 7:** Model summary.

Model	R	R Square	Adjusted R Square	Std. Error of the Estimate	Durbin–Watson
1	0.746	0.557	0.538	0.46977	1.960

**Table 8:** Coefficients of prediction model for online consumer behavior.

Model		Unstandardized Coefficients		Standardized Coefficients	t	*p*	95.0% Confidence Interval for B		Collinearity Statistics	
		B	SE	Beta			Lower Bound	Upper Bound	Tol.	VIF
1	(Constant)	−1.210	0.410		−2.949	0.004	−2.021	−0.399		
	Behavioral and attitudinal factors	0.348	0.133	0.267	2.616	0.010	0.085	0.611	0.301	3.322
	Social and peer influences	0.380	0.124	0.273	3.058	0.003	0.134	0.626	0.393	2.544
	Marketing and advertising impact	0.191	0.064	0.184	2.956	0.004	0.063	0.318	0.810	1.235
	Online experience	0.131	0.088	0.091	1.497	0.137	−0.042	0.304	0.859	1.164
	Brand-related factors	−0.008	0.084	−0.008	−0.089	0.929	−0.174	0.159	0.434	2.307
	Gen Z characteristics	0.205	0.070	0.198	2.928	0.004	0.067	0.343	0.685	1.461

**Table 9:** Moderation analysis summary of “brand-related factors and online consumer behavior”—perceived risk.

Model	R	R^2^	MSE	F	df1	df2	*p*
Brand-related factors	0.5071	0.2572	0.2522	34.3876	3	298	<0.001

**Table 10:** Regression coefficients of “brand-related factors and online consumer behavior”—perceived risk.

Predictor Variables	β	SE	t	*p*	LLCI	ULCI	VIF
Constant	3.5841	1.0764	3.3298	0.001	1.4658	5.7024	-
Brand-related factors (BRFs)	−0.2374	0.2953	−0.8042	0.4219	−0.8185	0.3436	1.000
Perceived risk (RISK)	−0.5083	0.3084	−1.6481	0.1004	−1.1151	0.0986	1.000
Interaction (BRF × RISK)	0.1956	0.0838	2.3336	0.0203	0.0306	0.3605	1.000

**Table 11:** Interaction test results of “brand-related factors and online consumer behavior”—perceived risk.

R^2^ Change	F	df1	df2	*p*
0.0136	5.4459	1	298	0.0203

**Table 12:** Conditional effects of BRFs at values of perceived risk to “online consumer behavior”.

RISK (Moderator Level)	Effect (B)	SE	t	*p*	LLCI	ULCI
Low (2.8566)	0.3212	0.0721	4.4552	<0.001	0.1793	0.4631
Moderate (3.4472)	0.4367	0.0505	8.6507	<0.001	0.3374	0.5361
High (4.0377)	0.5522	0.0693	7.9725	<0.001	0.4159	0.6886

**Table 13:** Moderation analysis summary of “Gen Z characteristics and online consumer behavior”—perceived risk.

Model	R	R^2^	MSE	F	df1	df2	*p*
1	0.5206	0.2711	0.3708	36.9406	3	298	<0.001

**Table 14:** Regression coefficients of “Gen Z characteristics and online consumer behavior”—perceived risk.

Predictor Variables	β	SE	t	*p*	LLCI	ULCI
Constant	3.7816	1.0116	3.7383	0.0002	1.7909	5.7724
Gen Z characteristics (GZCs)	−0.2451	0.2975	−0.8240	0.4106	−0.8305	0.3403
Perceived risk (RISK)	−0.5753	0.2874	−2.0021	0.0462	−1.1408	−0.0098
Interaction (GZC × RISK)	0.2110	0.0830	2.5434	0.0115	0.0478	0.3743

**Table 15:** Interaction test results of “Gen Z characteristics and online consumer behavior”—perceived risk.

R^2^ Change	F	df1	df2	*p*
0.0158	6.4690	1	298	0.0115

**Table 16:** Conditional effects of GZCs on values of perceived risk in terms of “online consumer behavior”.

RISK (Moderator Level)	Effect (B)	SE	t	*p*	LLCI	ULCI
Low (2.8566)	0.3578	0.0779	4.5947	0.0000	0.2045	0.5110
Moderate (3.4472)	0.4824	0.0551	8.7483	0.0000	0.3739	0.5909
High (4.0377)	0.6071	0.0694	8.7422	0.0000	0.4704	0.7437

## Data Availability

The data presented in this study are available on request from the corresponding author.
